# Social contact and the perceived impact of social distancing on health outcomes during the COVID-19 pandemic among community dwelling older adults taking part in the OPAL cohort study

**DOI:** 10.1186/s12889-024-18950-8

**Published:** 2024-06-05

**Authors:** Esther Williamson, Ioana R Marian, Paul Newell, Alana Morris, Mandy Slark, Sarah Lamb

**Affiliations:** 1 Nuffield Department of Rheumatology, Orthopaedics and Musculoskeletal Sciences, University of Oxford, Oxford, UK; 2 Oxford Clinical Trials Research Unit, Centre for Statistics in Medicine, Nuffield Department of Orthopaedics, Rheumatology and Musculoskeletal Sciences, University of Oxford, Oxford, UK; 3 Department of Public Health and Sports Sciences, Faculty of Health and Life Sciences, University of Exeter, Exeter, UK; 4 Centre for Rehabilitation Research, Nuffield Department of Orthopaedics, Rheumatology and Musculoskeletal Sciences, University of Oxford, Botnar Research Building, Windmill Road, Oxford, OX3 7LD UK

**Keywords:** COVID-19 pandemic, Physical health, Mental health, Older people, Social contact

## Abstract

**Background:**

During the COVID-19 pandemic, social distancing and reduced social contact may have affected older adults’ health.

**Objectives:**

To evaluate the perceived impact of social distancing on older adults’ health and explore the association between social contact and health outcomes.

**Design:**

Cross-sectional and longitudinal analyses of the OPAL cohort study.

**Subjects:**

Community dwelling older adults.

**Methods:**

We sent questionnaires to participants of an existing cohort study (*n* = 4328). Questions included the amount and type of social contact, and how often they went outside. Participants rated the impact of social distancing on their health. Sociodemographic factors and quality of life were available from previous questionnaires. We examined quality of life prior to and during the pandemic and explored the cross-sectional relationship between social contact and health using logistic regression.

**Results:**

There were 3856/4328 (89%) questionnaires returned. EQ-5D scores changed little compared to pre-pandemic scores but 25% of participants reported their overall health had worsened. The telephone was the most used method of contact (78%). Video calls were used least with 35% of participants not using them or having no access to them. 13% of respondents never went outside. Lower levels of contact were associated with increased risk of reporting worse health (Odds ratio (OR) 1.04 (95% CI 1.01–1.08)). Those experiencing financial strain and who spent less time outside experienced the largest increase in risk of reporting perceived worsened overall health. Those reporting a strain to get by financially were 4 times more likely to report worsened health than those who described themselves as quite comfortably off (OR 4.00 (95% CI 1.86–8.16)). Participants who reported never going outside were twice as likely to report worsened health compared to those who went outside daily (OR 2.00 (95% CI 1.57–2.54)).

**Conclusions:**

Less contact with other people was associated with perceived worsening in overall health. Although many older people reported using online technology, such as video calls, a substantial proportion were not using them. Older people facing financial strain were more likely to report worsened health, highlighting the impact of social inequalities during the pandemic. Going outside less was also associated with perceived worsened health.

**Supplementary Information:**

The online version contains supplementary material available at 10.1186/s12889-024-18950-8.

## Background

During the COVID-19 pandemic, stay-at-home orders were issued to reduce the spread of the COVID-19 virus. After stay-at-home orders were lifted, people were still asked to maintain social distancing from family, friends, and the wider community. Social distancing is described as a “public health practice that aims to prevent sick people from coming in close contact with healthy people in order to reduce opportunities for disease transmission” [1]. In the United Kingdom (UK), people with pre-existing health conditions making them vulnerable to COVID-19 were asked to shield by their General Practitioner. Shielding meant severely limiting contact with all other people and not leaving the house, even to exercise or shop.

Older adults may have been disproportionally affected by the impact of social distancing [2]. Many older adults rely on face-to-face interactions for social contact and, prior to the pandemic, many were not used to connecting digitally with friends and family, potentially leading to isolation and loneliness [3]. Loneliness is associated with worsening depression and anxiety [4] and declining mental health [5]. Surveys report that many older people felt lonelier during the pandemic [4, 6–8]. The use of technology has been proposed as a way to overcome barriers to social interaction for older adults [9] and increased use of technology was reported by older adults during the pandemic [10]. However, the impact of the use of these types of technology on the perceived health of older people during the pandemic is unclear.

Stay at home orders and social distancing also had the potential to impact on physical activity levels. Physical activity helps to delay the loss of physical function and reduce the risk of falling in older adults [11]. During the pandemic, reduced physical activity among older people was reported [5–7, 12, 13] but little has been reported on the impact on physical function including the ability to walk and to carry out usual activities.

The Oxford Pain, Activity and Lifestyle (OPAL) cohort study is a prospective cohort study of community dwelling older adults in England, UK and is broadly representative of the UK population [14]. This cohort provided an opportunity to study the self-perceived impact of social distancing during the COVID-19 pandemic on the health of older adults, and to investigate the type and frequency of contact during this time.

This study aims to (1) evaluate the impact of social distancing on an older person’s perceptions of their health, (2) describe the type and frequency of contact older adults had with people outside their homes and (3) explore the associations between social contact and other variables (such as being asked to shield, experiencing COVID-19 symptoms, going outside) and the perceived impact on participants’ overall health.

## Methods

### Study population

We recruited 5409 older adults to the OPAL cohort study [14]. People registered with a general practice, aged ≥ 65 years, and living in the community were eligible for invitation. Between October 2016 and March 2018, eligible participants were identified from electronic record searches of 35 general practice lists and a random sample of approximately 400 individuals was selected for invitation per practice. The sample was stratified by age (65–74 and 75 years and over). General practices were selected to ensure a geographical spread including urban and rural areas across England to capture diversity in both socioeconomic and ethnic profiles. Approximately 42% of those invited participated in the study (5409/12,839). Follow up postal questionnaires were sent yearly with Year 1 being sent between October 2017 and April 2019 and Year 2 being sent between November 2018 and March 2020. This nested study of the impacts of the COVID-19 pandemic was undertaken in the third year of OPAL cohort study follow up which began in December 2019.There were 4328/5409 (80% of those enrolled at baseline) participants retained in the cohort at this point.

The London - Brent Research Ethics Committee (16/LO/0348) approved the OPAL cohort study on the 10th of March 2016. All participants provided written informed consent.

### Data collection

We sent the COVID-19 questionnaire to 4328 participants The UK government issued strict “stay at home” orders on 23 March 2020 until 1 June 2020 when social distancing rules remained in place. Questionnaires were sent during June and July 2020. They were sent to participants as an additional OPAL study questionnaire (*n* = 2811/4328) or as part of their Year 3 follow up questionnaire (*n* = 1517/4328) depending on when their Year 3 follow up was due.

We collected data on whether participants were asked to shield, if they had been diagnosed and/or experienced symptoms of COVID-19, and any related hospital stays, changes in living arrangements, and whether they experienced bereavement of a close relative or friend. Social contact was measured by self-reported frequency of different types of contact with people outside their home (face-to-face; telephone calls; email, text or messaging; videocalls). Each item was rated on a six-point scale constructed with responses ranging from daily to never. We asked respondents to indicate if they did not have access to a phone, email, text or messaging and videocalls. The questions sent to participants are available in the supplementary materials.

Participants were asked to rate their perceived impact of social distancing on different aspects of their health (walking; ability to perform self-care and usual activities; pain and discomfort; mental health (such as feeling anxious or depressed); how connected they felt to family, friends, neighbours and society; ability to experience companionship; sleeping; and overall health). The questions were based on the categories of the EQ-5D-5 L [15]. We also created questions based on two questionnaires used to measure loneliness and social connectedness (Social Connectedness Scale [16] and Three-Item Loneliness Scale [17]). We used a seven-point scale constructed for the study with responses ranging from much worse to much better. We asked participants how often they went outside their own home (to shop or to exercise) during this time. (See Supplementary Materials for the questions used). Health-related quality of life was measured using the EQ-5D-5 L [15].

Sociodemographic information (age, sex, number of comorbidities, marital status, living arrangements, Index of Multiple Deprivation (IMD)) was available from the OPAL baseline questionnaire. The IMD is the official measure of relative deprivation which ranks every area in England from most deprived to least deprived based on seven domains (income, employment, education, health, crime, access to housing and services and living environment) [18]. Health-related quality of life data (EQ-5D-5 L) was available from the baseline, Year 1, and Year 2 questionnaires.

### Data analysis

We summarised data using means and standard deviations (SD), median and interquartile range (IQR) or counts and percentages (depending on data).

We estimated the associations between type and frequency of contact and each aspect of health using Spearman’s rank correlation coefficient. Preliminary analyses showed no meaningful associations between type and frequency of contact and health outcomes (See Supplementary Materials Table S1), so we created a new variable (social isolation score). This variable reflected the degree of social isolation experienced by a participant based on the total amount of contact that each participant had with people outside their home. We created the variable by summing the scores from each type of contact (face-to-face, telephone, texts/messages, videocalls scoring 0 = daily/a few times a week; 1 = once a week/a few times a month; 2 = once per month; 3 = never/no access). This produced a maximum score of 12 with a higher score indicating greater social isolation (less contact).

We conducted multivariable logistic regression to determine the association between the total amount of contact with other people outside their home (social isolation score) and the perceived impact on their health (dependent variable). We were primarily interested in identifying factors related to a worsening of perceived health. Therefore, participant responses to the question asking them to rate the perceived impact of social distancing on their overall health was categorised as a binary response (improved/same or worse). We estimated the odds ratio for each independent variable of participants responding to be worse.

We wanted to understand this association in relation to other variables with the potential to influence outcomes. Therefore, the model also included the following co-variates, covering a range of constructs including socioeconomic factors and factors related to their experiences of the COVID-19 pandemic.

- Age (continuous variable).
- Sex (Male/Female).
- IMD: participants were grouped into quintiles based on the IMD of their home address (Q1 = least deprived to Q5 = most deprived).
- Adequacy of income (Quite comfortably off/Manage without much difficulty/Careful with money/A strain/Prefer not to say).
- Baseline relationship status (Married/Living with partner/Never married/Separated or divorced/Widowed).
- Lived alone (Yes/No).
- Asked to shield (Yes/No).
- Experienced symptoms of COVID-19 (Yes/No).
- Experienced a bereavement due to COVID-19 (Yes/No).
- How often they went outside (Daily or a few times a week/Weekly or a few times a month/Once per month/Never).

The Akaike Information Criterion (AIC) was used to select the variables in the model.

We examined quality of life scores (EQ-5D-5 L) over time using data from the baseline, Year 1, Year 2, and the COVID-19 questionnaires, using a mixed effects linear regression model adjusted for age, sex, baseline score, days since baseline, and whether it was completed during the pandemic or not, and with random effects for participants. EQ-5D-5 L index values were calculated by applying the UK cross-walk mapping [19].

## Results

There were 3856/4328 (89%) questionnaires returned (71% of those enrolled at baseline). Of those who did not respond, 50/4328 (1.2%) participants had died. The mean age of the cohort when they completed the COVID-19 questionnaire was 77 years (SD 6.1, range 67 to 100 years old). A description of respondents is provided in Table 1.

**Table 1:** Demographic data, prevalence of COVID-19 and other related variables (*n* = 3856)

Variables	Number (%) or mean (SD)
Age (years)	77.0 (6.1), range 67 to 100
Sex^§^ Female	1,967 (51.0%)
Baseline EQ-5D-5 L (*n* = 3810) ^§^	0.798 (0.18)
Year 2 EQ-5D-5 L ^Ω^	0.789 (0.19)
Covid-19 Questionnaire EQ-5D-5 L	0.770 (0.20)
Change in EQ-5D-5 L scores from Year 2 to Covid-19 Questionnaire (*n* = 3645)	− 0.018 (0.13)
Number of comorbidities^§^ None	498 (12.9%)
1	1153 (29.9%)
2	1176 (30.5%)
3 or more	1029 (26.7%)
Index of Multiple Deprivation^§^ Quintiles – Q1 (least deprived)	338 (8.8%)
Q2	432 (11.2%)
Q3	842 (21.8%)
Q4	864 (22.4%)
Q5 (most deprived)	1380 (35.8%)
Adequacy of Income^§^ Quite comfortably off	1430 (37.1%)
(missing data *n* = 19, < 1%) Get by without much difficulty	1430 (37.1%)
Have to be careful with money	731 (19.0%)
Find it a strain to get by	37 (1.0%)
Prefer not to say	231 (6.0%)
Relationship status^§^ (missing data *n* = 6, < 1%) Married	2,553 (66.2%)
Living with partner	148 (3.8%)
Never married	156 (4.1%)
Separated or divorced	310 (8.0%)
Widow/widower	683 (17.7%)
Living alone^§^ (missing data *n* = 12, < 1%) Yes	1022 (26.5%)
No	2822 (73.2%)
Asked to shield (missing data *n* = 50, 1.3%) Yes	651 (16.9%)
No	3155 (81.8%)
Experienced COVID-19 symptoms (missing data *n* = 359, 9.3%) Yes	179 (4.6%)
No	3642 (94.4%)
COVID-19 diagnosed by doctor (missing data *n* = 43, 1.1%) Yes	35 (< 1%)
No	3778 (98.0%)
Had a COVID-19 test (missing data *n* = 53, 1.4%) Yes	451 (11.7%)
No	3352 (86.9%)
COVID-19 test results (missing data *n* = 7/451, 1.6%) Negative	404/451 (89.6%)
Positive	7/451 (1.6%)
Unknown	33/451 (7.3%)
COVID-19 hospital admission Yes	10 (< 1%)
(Missing data *n* = 255, 6.2%) No	3591 (93.3%)
Length of hospital stay* (*n* = 10)	3 [1, 6]
Needing assistance with breathing while hospitalised Yes	2/10 (20%)
(Missing data *n* = 1/10, 10%) No	7/10 (70%)
Changes in living arrangements due to COVID-19 Yes	130 (3.4%)
(missing data *n* = 232, 6.0%) No	3494 (90.6%)
Bereavement of close family member or friend due to COVID-19 Yes	177 (4.6%)
(missing data *n* = 106, 2.7%) No	3573 (92.7%)

^§^ From OPAL baseline questionnaire; ^Ω^ from OPAL Year 2 questionnaire; *Median (IQR)

Normative data Eq. 5D-5 L for an English Population: Female: 65–69 years = 0.775 (0.770–0.795); 70–74 years = 0.784 (0.779–0.801); 75–79 years = 0.730 (0.724–0.755); 80–84 years = 0.710 (0.699–0.733); 85–89 years = 0.666 (0.657–0.707); 90 + years = 0.666 (0.651–0.721); Male: 65–69 years = 0.797 (0.792–0.818); 70–74 years: 0.801 (0.794–0.818); 75–79 years = 0.788 (0.781–0.806); 80–84 years = 0.767 (0.760–0.801); 85–89 years = 0.727 (0.704–0.764); 90 + years = 0.656 (0.635–0.730) [20]

**Fig. 1:**
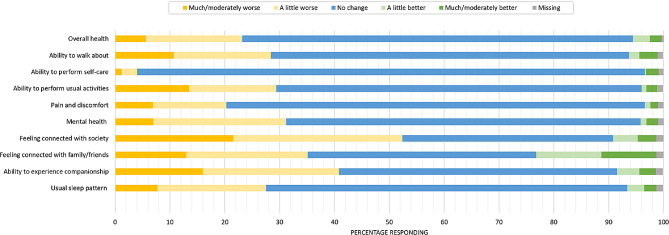
The perceived impact of social distancing on participants’ physical and mental health (*n* = 3856)

The perceived impact of social distancing on participants’ health is presented in Fig. 1 and Supplementary Materials Table S2.

Just over 70% (2713/3856) of participants did not perceive any change in their overall health due to social distancing, and approximately 5% (203/3856) reported improvements. Nearly a quarter (895/3856) of participants reported worsening of overall health, albeit most reported it to be a little worse. 11% (416/3856) of participants reported their ability to walk about as being moderately or much worse. We observed similar changes in reported ability to perform usual activities (14%; 523/3856). Ability to perform self-care was the least affected, with 93% (3571/3856) reporting no change.

Three-quarters (2944/3856) of participants reported no change in the pain or discomfort that they usually experienced, but around 20% (782/3856) reported a worsening of their usual pain and discomfort. As well as impacting on physical health, nearly a third (1201/3856) of participants reported worsening of mental health. Connections with friends and family as well as society were impacted. Many reported worsening of connections with family and friends (around 35%, 1357/3856) but 22% (841/3856) of participants also reported improvements, with 10% (383/3856) stating these were moderately better to much better. Over half (2023/3856) of the participants reported a worsening of their connections with society due to social distancing. Likewise, ability to experience companionship was affected, with 40% (1576/3856) reporting this as worse. Usual sleep patterns were affected with 28% (1064/3856) of participants reporting this to be worse.

**Fig. 2:**
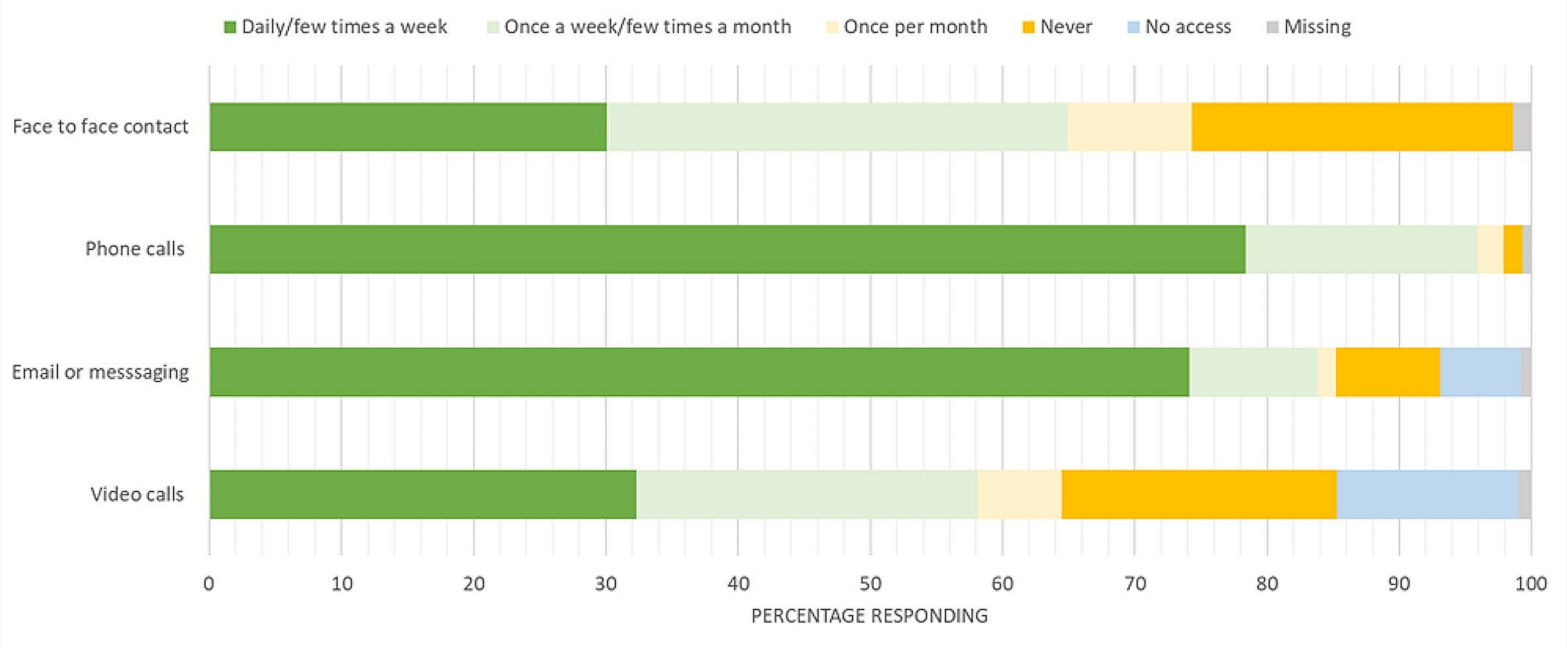
Type and frequency of contact with people outside of participants’ homes (*n* = 3856)

We present the type and frequency of contact with people outside participants’ homes in Fig. 2 and Supplementary Materials Table S3.

Around 30% (1160/3856) of the participants had face to face contact with someone from outside their home daily or a few times a week, but 25% (936/3856) of participants reported never having any face-to-face contact. The most used method of communication was phone calls, used by 78% (3021/3856) of participants on a frequent basis (daily or a few times a week). Email or messaging were used by 74% (2858/3856) of participants at least a few times a week. However, 7.9% (303/3856) and 6.1% (235/3856) of participants, respectively, reported never using or had no access to these types of communication. Video calls were the least used, with about a third (1246/3856) of participants using them at least a few times a week. Approximately 21% (803/3856) of participants never used them and 13.7% (530/3856) had no access.

The median social isolation score was 3 (IQR 0–12; higher score = less contact). When separated into quartiles, 827/3856 (21.9%) scored in the highest quartile (Q4) indicating greater social isolation and less contact with other people. Nearly 30% scored in the lowest quartile (Q1) (1137/3856) indicating higher levels of contact with other people and less social isolation. There were 1131/3856 (29.3%) in Q2 and 737/3856 (19.1%) in Q3.

How often people reported that they went outside their home is reported in Table 2.

**Table 2:** Frequency of going outside the home (*n* = 3856)

Frequency of going outside	Number of participants (%)
**Daily**	1304 (33.8%)
**A few times a week**	1034 (26.8%)
**Once a week**	547 (14.2%)
**A few times a month**	259 (6.7%)
**Once per month**	161 (4.2%)
**Never**	516 (13.4%)
***Missing***	**35 (0.9%)**

The multivariable logistic regression analyses are reported in Table 3. A higher social isolation score (less contact) was associated with a small increase in the odds of reporting a worse overall health. Older age, being female and living alone were also associated with increased odds of worse outcome. Participants were more likely to report worse overall health if they had experienced symptoms or had been diagnosed with COVID-19, been asked to shield, or reported a bereavement of close family or friend due to COVID-19. The participant’s rating of adequacy of their income was the most strongly associated variable with increased odds of reporting worse overall health. Going outside less frequently was also associated with a substantial increase in reporting worse overall health.

Quality of life scores (EQ-5D-5 L) over time are presented in the Supplementary Materials (Figure S2). The mean time from baseline data collection to follow up EQ-5D data collection was 366 days (SD 28 days) for Year 1, 739 days (SD 32) for Year 2 and 1063 days (SD 159) for the COVID-19 questionnaire. There was no statistically significant change in EQ-5D-5 L scores over time (*p* = 0.09).

**Table 3:** Multivariable logistic regression analyses to identify factors associated with a response of worse overall health due to the impact of social distancing (Odds ratios (95% Confidence Intervals))

Independent variables	Impact on overall health	*P* value
**Social Isolation Score** ^**Ω**^	1.04 (1.01, 1.08)	0.02
**Age (years)**	1.02 (1.01, 10.4)	0.00
**Sex** Male	Reference	
Female	1.21 (1.02, 1.43)	0.03
**Index of multiple deprivation∞** Least deprived Q1	Reference	
Q2	1.05 (0.75, 1.48)	0.78
Q3	0.85 (0.52, 1.16)	0.31
Q4	0.72 (0.52, 0.99)	0.04
Most deprived Q5	0.81 (0.60, 1.09)	0.17
**Adequacy of income** Quite comfortably off	Reference	
Manage without much difficulty	1.28 (1.05, 1.56)	0.02
Careful with money	2.14 (1.71, 2.69)	0.00
A strain	4.00 (1.86, 8.16)	0.00
Prefer not to say	1.18 (0.82, 1.71)	0.38
**Living alone** No	Reference	
Yes	1.53 (1.03, 2.28)	0.04
**Relationship status** Married	Reference	
Living with partner	0.82 (0.53, 1.28)	0.39
Never Married	1.07 (0.63, 1.80)	0.81
Separated or divorced	0.92 (0.58, 1.46)	0.73
Widow/widower	0.67 (0.44, 1.01)	0.06
**Asked to shield** No	Reference	
Yes	1.72 (1.40, 2.10)	0.00
**COVID-19 symptoms** No	Reference	
Yes	2.23 (1.59, 3.14)	0.00
**Bereavement**^**β**^: No	Reference	
Yes	1.45 (1.02, 2.06)	0.04
**Went outside home**		
Daily/few times a week	Reference	
Weekly/ A few times a month	1.56 (1.27, 1.91)	0.00
Once per month	2.64 (1.84, 3.78)	0.00
Never	2.00 (1.57, 2.54)	0.00

Ω 0 to 12, where 12 is great social isolation (less contact); β Bereavement of close family or friend due to COVID-19

## Discussion

We provide a snapshot of the experience of participants during the early stage of the COVID-19 pandemic. Around one-quarter of participants reported a worsening of their health due to the impact of social distancing. Participants were in contact with people outside their households using different types of communication methods. Around 30% had face to face contact with people. The majority communicated using the telephone. The least used method was video calls and over 30% of participants did not use them or had no access. This is consistent with data reported by Ofcom, who reported that 61% of older adults made at least one video call each week in May 2020 (having increased from 22% in February 2020).

Many participants felt less connected to family, friends and society and less able to experience companionship. Lack of connectedness can contribute to loneliness with negative consequences for health-related quality of life in older adults and may explain the impact on general health [21–23]. However, there was also a proportion who felt more connected during this time and a proportion who rated their health outcomes as improved. Other studies have described how some older adults were able to create or identify positive impacts of the pandemic (referred to as silver linings) when under social distancing or stay at home mandates including more meaningful time with loved ones or enjoying new hobbies [24]. Our data also reflects “silver linings” for some participants demonstrating the variety of experiences of older adults during the pandemic.

When we examined health-related quality of life over time using pre-pandemic data, there was very little change, with differences being less than published values for a minimally important difference in the EQ-5D [25]. This contrasts with other findings. The questions asking participants about the impact of social distancing were dependent on recall of their previous health state, while the EQ-5D is less affected by this as it related to their health state today which may explain the difference.

We found no meaningful associations between the frequency of specific types of contact and perceived health changes, but overall lower amounts of contact were associated with increased odds of reporting worsened overall health. This may suggest that the type of contact was less important than the amount.

Other variables showed stronger relationships with the chance of reporting worsened overall health. There were three factors which at least doubled the risk of reporting a worse health outcome. The first factor was reporting symptoms or being diagnosed with COVID-19. The longer-term impact of the COVID-19 virus has been well documented which may be reflected in these findings (for example [26]). Secondly, those who went outside less frequently were also more likely to report worsening health. Going outside encourages physical activity [27] although we did not ask about the activity done while outside. Regular physical activity contributes to better health in older people [28]. It is possible that benefits were derived from being outside. Greater exposure to green spaces such as parks and woodlands has been associated with better health in older people [27]. Having access to such areas appeared beneficial to older people during the pandemic and this should not be forgotten now the pandemic has passed [27]. This may be another factor contributing to health inequalities as people living in low socioeconomic areas have fewer green spaces in their neighbourhoods [27].

Finally, the strongest association was related to adequacy of income. Participants reporting it was a strain to get by were four times more likely to report worsened overall health compared to people who were comfortably off. This response (a strain to get by) was only given by a small number of participants (1% of respondents) resulting in wide confidence intervals indicating some uncertainty in this finding. However, those needing to be careful with money (19% of respondents) were more than twice as likely to report worsened overall health compared to people who were comfortably off (with much smaller confidence intervals) supporting the association between lower ratings of perceived adequacy of income and perceived health. The links between social determinants of health (which encompasses income/wealth, economic stability, education and employment) and health outcomes are well documented, and this was evident during the pandemic [29]. However, we included another variable measuring a socioeconomic construct, the IMD, which was not associated with a report of worsened overall health. This may be due to the differences in the measures. The IMD covers seven domains of deprivation, not just income, also taking into account employment, education, health, crime, access to housing and services, and living environment [30, 31]. It reflects the socioeconomic situation of the area in which an individual lives rather than specific to them. In contrast, the perceived adequacy of income questions measures the individual participant’s perceptions about their income and whether it is sufficient for their needs [32]. Adequacy of income has been linked to health outcomes in other studies (for example [33]), and is considered a robust indicator of financial capacity in older age [32].

### Strengths and potential limitations

A strength to this study is that we used an established cohort study to undertake this research. The response rate from those retained in the cohort at three years follow up was high (89%) and the cohort has good representativeness to the general population [14]. We also wanted to collect data while participants were experiencing social distancing mandates. Questionnaires were sent after the strict “stay at home” orders were lifted but stringent social distancing recommendations were still in place, so we achieved this.

There are some potential limitations. This study relied on participants’ perception of their health using single questions for each domain. We did not use a validated questionnaire but the questions asked were informed by categories within the EQ-5D-5 L [15] and questionnaires used to measure loneliness and social connectedness [16, 17]. We also worked with patient representatives to ensure the questions had face validity. More comprehensive and validated measures may have resulted in different findings. Recall bias may also have influenced findings. Participants had to remember the types and frequency of contact they had with other people. We also did not collect information about who they were in contact with or how long they spent together. Also, when we created the variable to reflect the overall contact with people outside their homes (social isolation score), we presumed that all forms of contact had the same value and that their frequency of use contributed value in the same way which may not be the case. Finally, this research was conducted at the start of the pandemic and is primarily cross-sectional in design, so we do not know the longer-term consequences.

### Implications and further research

This study demonstrated that more social contact was associated with better perceived health outcomes. Digital technology can facilitate social contact and reduce isolation in older adults [9] but many older adults lack the skills to access these technologies or may experience barriers to access, including affordability [34]. There is a need to understand how to support older adults in their use of digital technology and to avoid widening the digital divide [34, 35]. This is especially important in the light of our findings that those reporting financial strain were at greater risk of reporting worsened health. Going outside less was also associated with worsened health so developing effective interventions to promote regular outdoor activity in older adults at both individual level and within the built environment should be considered.

In conclusion, less contact with other people was associated with perceived worsening in overall health, but the type of contact did not matter. Although many older people reported using online technology, such as video calls, a substantial proportion were not using them. Older people facing financial strain were more likely to report worsened health, highlighting the impact of social inequalities during the pandemic. Going outside less was also associated with perceived worsened health.

### Electronic supplementary material

Below is the link to the electronic supplementary material.

Supplementary Material 1

## Data Availability

The data and study materials are available from the Chief Investigator, Professor Sallie Lamb (s.e.lamb@exeter.ac.uk) upon reasonable request.

## Abbreviations

AIC: Akaike Information Criterion
CI: Confidence Intervals
EQ-5D: EuroQol- 5 Dimension
IMD: Index of Multiple Deprivation
IQR: Interquartile Range
OPAL: Oxford Pain Activity and Lifestyle

## References

[1] Pearce K. What is social distancing and how can it slow the spread of COVID-19? Baltimore, Maryland USA: John Hopkins University; 2020 [ https://hub.jhu.edu/2020/03/13/what-is-social-distancing/.

[2] Brooke J, Jackson D. Older people and COVID-19: isolation, risk and ageism. J Clin Nurs. 2020. 10.1111/jocn.15274 32239784

[3] Petretto DR, Pili R. Ageing and COVID-19: what is the role for Elderly people? Geriatr (Basel). 2020;5(2). 10.3390/geriatrics5020025 PMC7345165 32357582

[4] Robb CE, de Jager CA, Ahmadi-Abhari S, Giannakopoulou P, Udeh-Momoh C, McKeand J (2020). Associations of Social isolation with anxiety and Depression during the early COVID-19 pandemic: a survey of older adults in London, UK. Front Psychiatry.

[5] Suzuki Y, Maeda N, Hirado D, Shirakawa T, Urabe Y (2020). Physical activity changes and its risk factors among Community-Dwelling Japanese older adults during the COVID-19 epidemic: associations with Subjective Well-Being and Health-Related Quality of Life. Int J Environ Res Public Health.

[6] Brown L, Mossabir R, Harrison N, Brundle C, Smith J, Clegg A. Life in lockdown: a telephone survey to investigate the impact of COVID-19 lockdown measures on the lives of older people (≥ 75 years). Age Ageing. 2020. 10.1093/ageing/afaa255 PMC7717141 33173949

[7] Emerson KG (2020). Coping with being cooped up: social distancing during COVID-19 among 60 + in the United States. Rev Panam Salud Publica.

[8] van Tilburg TG, Steinmetz S, Stolte E, van der Roest H, de Vries DH. Loneliness and mental health during the COVID-19 pandemic: a study among Dutch older adults. J Gerontol B Psychol Sci Soc Sci. 2020. 10.1093/geronb/gbaa111 PMC7454922 32756931

[9] Sen K, Prybutok G, Prybutok V (2022). The use of digital technology for social wellbeing reduces social isolation in older adults: a systematic review. SSM Popul Health.

[10] Sixsmith A, Horst BR, Simeonov D, Mihailidis A. Older People’s Use of Digital Technology During the COVID-19 Pandemic. Bull Sci Technol Soc. 42: © The Author(s). 2022.; 2022. pp. 19–24. 10.1177/02704676221094731 PMC9038938 38603230

[11] Dipietro L, Campbell WW, Buchner DM, Erickson KI, Powell KE, Bloodgood B (2019). Physical activity, Injurious Falls, and physical function in aging: an Umbrella Review. Med Sci Sports Exerc.

[12] Carriedo A, Cecchini JA, Fernandez-Rio J, Méndez-Giménez A (2020). COVID-19, Psychological Well-being and physical activity levels in older adults during the Nationwide Lockdown in Spain. Am J Geriatr Psychiatry.

[13] Visser M, Schaap LA, Wijnhoven HAH. Self-reported impact of the COVID-19 pandemic on Nutrition and Physical Activity Behaviour in Dutch older adults living independently. Nutrients. 2020;12(12). 10.3390/nu12123708 PMC7760336 33266217

[14] Sanchez Santos MT, Williamson E, Bruce J, Ward L, Mallen CD, Garrett A (2020). Cohort profile: Oxford Pain, Activity and Lifestyle (OPAL) Study, a prospective cohort study of older adults in England. BMJ Open.

[15] Herdman M, Gudex C, Lloyd A, Janssen M, Kind P, Parkin D (2011). Development and preliminary testing of the new five-level version of EQ-5D (EQ-5D-5L). Qual Life Res.

[16] Lee RM, Draper M, Lee S (2001). Social connectedness, dysfunctional interpersonal behaviors, and psychological distress: testing a mediator model. J Couns Psychol.

[17] Hughes ME, Waite LJ, Hawkley LC, Cacioppo JT (2004). A short scale for measuring loneliness in large surveys: results from two Population-Based studies. Res Aging.

[18] Statistics N. English indices of deprivation 2015. In: Ministry of Housing CLG, editor. England2015.

[19] van Hout B, Janssen MF, Feng YS, Kohlmann T, Busschbach J, Golicki D (2012). Interim scoring for the EQ-5D-5L: mapping the EQ-5D-5L to EQ-5D-3L value sets. Value Health.

[20] McNamara S, Schneider PP, Love-Koh J, Doran T, Gutacker N (2023). Quality-adjusted life expectancy norms for the English Population. Value Health.

[21] Ong AD, Uchino BN, Wethington E (2016). Loneliness and health in older adults: a Mini-review and Synthesis. Gerontology.

[22] Tan SS, Fierloos IN, Zhang X, Koppelaar E, Alhambra-Borras T, Rentoumis T et al. The Association between Loneliness and Health Related Quality of Life (HR-QoL) among Community-Dwelling Older citizens. Int J Environ Res Public Health. 2020;17(2). 10.3390/ijerph17020600 PMC7013468 31963427

[23] Choi EY, Farina MP, Zhao E, Ailshire J. Changes in social lives and loneliness during COVID-19 among older adults: a closer look at the sociodemographic differences. Int Psychogeriatr. 2023:1–13. 10.1017/S1041610222001107 PMC10198802 36621851

[24] Wilder J, Lauderdale DS, Hawkley L (2023). Did Resilience and Socioeconomic Status Predict older adults’ finding a silver lining in COVID?. Innov Aging.

[25] McClure NS, Sayah FA, Xie F, Luo N, Johnson JA (2017). Instrument-defined estimates of the minimally important difference for EQ-5D-5L index scores. Value Health.

[26] Subramanian A, Nirantharakumar K, Hughes S, Myles P, Williams T, Gokhale KM (2022). Symptoms and risk factors for long COVID in non-hospitalized adults. Nat Med.

[27] Levinger P, Cerin E, Milner C, Hill KD (2022). Older people and nature: the benefits of outdoors, parks and nature in light of COVID-19 and beyond- where to from here?. Int J Environ Health Res.

[28] Bailey L, Ward M, DiCosimo A, Baunta S, Cunningham C, Romero-Ortuno R et al. Physical and Mental Health of Older People while Cocooning during the COVID-19 Pandemic. Qjm. 2021. 10.1093/qjmed/hcab015 PMC7928635 33471128

[29] Green H, Fernandez R, MacPhail C (2021). The social determinants of health and health outcomes among adults during the COVID-19 pandemic: a systematic review. Public Health Nurs.

[30] Ministry of Housing C, Local G. English indices of deprivation 2019. Index of multiple deprivation [Internet]; 2019.

[31] McLennan D, Noble S, Noble M, Plunkett E, Wright G, Gutacker N. The English Indices of Deprivation 2019: Technical Report. In: Ministry of Housing CaLG, editor. London2019.

[32] Litwin H, Sapir EV (2009). Perceived income adequacy among older adults in 12 countries: findings from the survey of health, ageing, and retirement in Europe. Gerontologist.

[33] Meisters R, Putrik P, Westra D, Bosma H, Ruwaard D, Jansen M (2023). Two sides of the same coin? Absolute income and perceived income inadequacy as social determinants of health. Int J Equity Health.

[34] Welch V, Ghogomu ET, Barbeau VI, Dowling S, Doyle R, Beveridge E (2023). Digital interventions to reduce social isolation and loneliness in older adults: an evidence and gap map. Campbell Syst Rev.

[35] Datta A, Bhatia V, Noll J, Dixit S (2019). Bridging the Digital divide: challenges in opening the Digital World to the Elderly, Poor, and digitally illiterate. IEEE Consum Electron Mag.

