# Supplementary material for: Social contact and the perceived impact of social distancing on health outcomes during the COVID-19 pandemic among community dwelling older adults taking part in the OPAL cohort study
Source: BMC Public Health. 2024 Jun 5;24:1510. doi: 10.1186/s12889-024-18950-8 (PMC11155085; doi:10.1186/s12889-024-18950-8)
Supplement: Supplementary file 1 — Supplementary Material 1 [file 12889_2024_18950_MOESM1_ESM.docx]

Supplementary materials:

Table S1 Correlation between type of contact and perceived impact on health

|  | Perceived impact on health^2^ | | | | | | | | | |
| --- | --- | --- | --- | --- | --- | --- | --- | --- | --- | --- |
| **Type of contact^1^** | **Impact on overall health** | **Impact on walking** | **Ability to perform self-care** | **Ability to perform usual activities** | **Pain & discomfort** | **Mental health** | **Feeling connected with family & friends** | **Feeling connected with society** | **Able to experience companionship** | **Usual sleep pattern** |
| **Face to face contact** | -0.0136 | 0.0009 | -0.0208 | -0.0179 | -0.0124 | -0.0144 | -0.0918*** | -0.0913*** | -0.0650*** | -0.0086 |
| **Phone calls** | -0.0328** | 0.0032 | -0.0232 | -0.0129 | -0.0001 | 0.0171 | -0.0627*** | -0.0086 | 0.0064 | 0.0099 |
| **Email, mobile phone text messages or messaging services**  **(e.g. What's App)** | -0.0825*** | -0.0365** | -0.0625*** | 0.0299* | -0.0547*** | 0.0091 | -0.0040 | 0.0589*** | 0.0571*** | -0.0015 |
| **Video calls**  **(e.g. Skype, Zoom, Facetime or What's App)** | -0.1014*** | -0.0562*** | -0.0646*** | 0.0159 | -0.0733*** | 0.0143 | 0.0240 | 0.0621*** | 0.0623*** | -0.0002 |

| *** p<.01, ** p<.05, * p<.1, Spearman’s Correlation  Notes:  Due to the scoring of variables, a negative correlation indicates that lower contact was related to poorer health |
| --- |

^1^Type of contact score range is 1 to 6 or 1 to 7, with higher scores indicating less contact/use

^2^Perceived impact on health score range is 1 to 7, with higher scores indicating a positive impact on health

Table S2: The perceived impact of social distancing on participants’ physical and mental health (n=3856)

|  | **Impact on overall health** | **Impact on walking** | **Ability to perform self-care** | **Ability to perform usual activities** | **Pain and discomfort** |
| --- | --- | --- | --- | --- | --- |
| **Much worse** | 44 (1.1%) | 125 (3.2%) | 4 (0.1%) | 190 (4.9%) | 84 (2.2%) |
| **Moderately worse** | 172 (4.5%) | 291 (7.5%) | 41 (1.1%) | 333 (8.6%) | 181 (4.7%) |
| **A little worse** | 679 (17.6%) | 683 (17.7%) | 109 (2.8%) | 612 (15.9%) | 517 (13.4%) |
| **No change** | 2713 (71.2%) | 2519 (65.3%) | 3571 (92.6%) | 2568 (66.6%) | 2944 (76.3%) |
| **A little better** | 118 (3.1%) | 73 (1.9%) | 9 (0.2%) | 36 (0.9%) | 37 (1.0%) |
| **Moderately better** | 43 (1.1%) | 50 (1.3%) | 20 (0.5%) | 24 (0.6%) | 20 (0.5%) |
| **Much better** | 42 (1.1%) | 78 (2.0%) | 68 (1.18%) | 52 (1.3%) | 35 (0.9%) |
| ***Missing*** | 45 (1.1%) | 37 (1.0%) | 34 (0.9%) | 41 (1.1%) | 38 (1.0%) |
|  | **Mental health** | **Feeling connected with family/friends** | **Feeling connected with society** | **Able to experience companionship** | **Usual sleep pattern** |
| **Much worse** | 57 (1.5%) | 125 (3.2%) | 262 (6.8%) | 210 (5.4%) | 74 (1.9%) |
| **Moderately worse** | 211 (5.5%) | 378 (9.8%) | 572 (14.8%) | 410 (10.6%) | 225 (5.8%) |
| **A little worse** | 933 (24.2%) | 854 (22.1%) | 1189 (30.8%) | 956 (24.8%) | 765 (19.8%) |
| **No change** | 2491 (64.6%) | 1609 (41.7%) | 1481 (38.4%) | 1954 (50.7%) | 2541 (65.9%) |
| **A little better** | 41 (1.1%) | 458 (11.9%) | 172 (4.5%) | 158 (4.1%) | 119 (3.1%) |
| **Moderately better** | 31 (0.8%) | 192 (5.0%) | 75 (1.9%) | 58 (1.5%) | 50 (1.3%) |
| **Much better** | 49 (1.3%) | 191 (5.0%) | 56 (1.5%) | 58 (1.5%) | 35 (0.9%) |
| ***Missing*** | 43 (1.1%) | 49 (1.3%) | 49 (1.3%) | 52 (1.3%) | 47 (1.2%) |

Table S3: Type and frequency of contact with people outside participants’ homes

| **Frequency** | **Face to face contact** | **Phone calls** | **Email, mobile phone text messages or messaging services**  **(e.g. What's App)** | **Video calls**  **(e.g. Skype, Zoom, Facetime or What's App)** |
| --- | --- | --- | --- | --- |
| **Daily** | 323 (8.4%) | 1280 (33.2%) | 1521 (39.4%) | 369 (9.6%) |
| **A few times a week** | 837 (21.7%) | 1741 (45.2%) | 1337 (34.7%) | 877 (22.7%) |
| **Once a week** | 826 (21.4%) | 346 (9.0%) | 169 (4.4%) | 565 (14.7%) |
| **A few times a month** | 517 (13.4%) | 327 (8.5%) | 204 (5.3%) | 429 (11.1%) |
| **Once per month** | 363 (9.4%) | 78 (2.0%) | 55 (1.4%) | 246 (6.4%) |
| **Never** | 936 (24.3%) | 53 (1.4%) | 303 (7.9%) | 803 (20.8%) |
| ***No access*** | N/A | 5 (0.1%) | 235 (6.1%) | 530 (13.7%) |
| ***Missing*** | 54 (1.4%) | 26 (0.7%) | 32 (0.8%) | 37 (1.0%) |

Figure S1 EQ5D5L scores over time


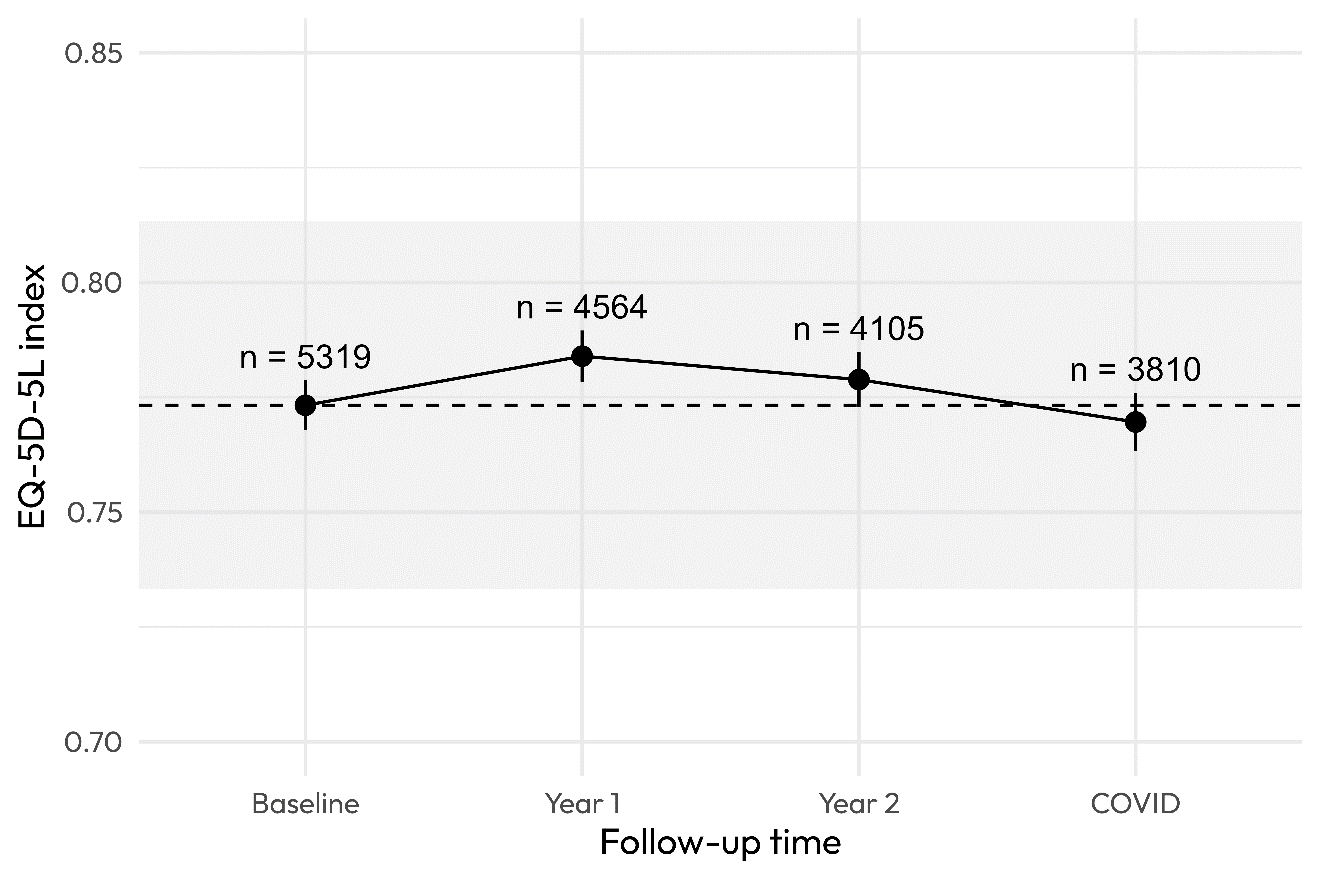


*Notes: The number of participants that died during the study: 1) Baseline to Year 1 = 61/5409 (1.1%); 2) Year 1 to Year 2 =120/5409 (2.2%); 3) Year 2 to Covid Questionnaire = 35/5409 (0.6%); 4) During Covid Questionnaire follow up (June 2020 to December 2020) = 50 (0.9%)*

*The shaded area represents the published minimally important difference in the EQ5D of 0.04 [20*

**The COVID-19 survey questions sent to OPAL participants:**

**Many people experienced changes in their lives due to the COVID-19 Pandemic. We would like to ask you about your experiences.**

1. Did you receive a letter from your GP saying you needed to take extra steps to protect yourself from COVID-19 (known as shielding)?

Responses: Yes / No

2. Have you experienced symptoms that you think were due to COVID-19 (such as a high temperature or new, continuous cough)?

Responses: Yes / No

3. Has a doctor told you that they thought you had COVID-19?

Responses: Yes / No

4. Were you given a test for COVID-19?

Responses: Yes / No

**If yes**, did this confirm that you had Covid-19?

Responses: Unknown/Yes / No

5. Were you admitted to hospital due to symptoms of COVID-19?

Responses: Yes / No

**If yes**, how many nights did you remain in hospital?

**If yes**, did you require mechanical assistance to breathe from a CPAP machine or ventilator?

Responses: Yes / No

6. Did you have to make changes to your living arrangements because of COVID-19 (such as move in with relatives/friends or have relatives/friends move in with you)?

Responses: Yes / No

7. Have you experienced any bereavement of a close family member or close friend due to COVID-19?

Responses: Yes / No

**The government asked everyone to stay home and away from all people who were not members of your household (known as social distancing) during the COVID-19 Pandemic. We would like to know about the type of contact you had with people outside your home (not people living with you in the same household) during this time. Please select one response.**

8. How often did you have face to-face contact with people outside your home (such as your family, nurse or carer)?

Responses: Daily/A few times a week/Once a month/A few times a month/once per month/Never

9. How often did you go outside your own home (to shop or to exercise)?

Responses: Daily/A few times a week/Once a month/A few times a month/once per month/Never

10. How often did you speak on the phone with people outside of your home?

Responses: Daily/A few times a week/Once a month/A few times a month/once per month/I never use/I have no access

11. How often did you have contact with people outside your home using Email, mobile phone text messages or other messaging services such as Messenger or What's App?

Responses: Daily/A few times a week/Once a month/A few times a month/once per month/I never use/I have no access

12. How often did you have contact with people outside your home using video calls such as Skype, Zoom, Facetime or What's App?

Responses: Daily/A few times a week/Once a month/A few times a month/once per month/I never use/I have no access

**We would like to understand how social distancing has impacted on different aspects of your life. Please select one response.**

13. Your ability to walk about

Responses: Much worse/Moderately worse/A little worse/No change/A little better/Moderately better/Much better

14. Your ability to perform self-care (e.g. washing or dressing)

Responses: Much worse/Moderately worse/A little worse/No change/A little better/Moderately better/Much better

15. Your ability to perform usual activities (e.g. work, study, housework, family or leisure activities)

Responses: Much worse/Moderately worse/A little worse/No change/A little better/Moderately better/Much better

16. Pain and discomfort

Responses: Much worse/Moderately worse/A little worse/No change/A little better/Moderately better/Much better

17. Your mental health (such as feeling anxious or depressed)

Responses: Much worse/Moderately worse/A little worse/No change/A little better/Moderately better/Much better

18. How connected you felt to family, friends and neighbours

Responses: Much worse/Moderately worse/A little worse/No change/A little better/Moderately better/Much better

19. How connected you felt with society

Responses: Much worse/Moderately worse/A little worse/No change/A little better/Moderately better/Much better

20. Your ability to experience companionship

Responses: Much worse/Moderately worse/A little worse/No change/A little better/Moderately better/Much better

21. Your usual sleep pattern

Responses: Much worse/Moderately worse/A little worse/No change/A little better/Moderately better/Much better

22. Your overall health

Responses: Much worse/Moderately worse/A little worse/No change/A little better/Moderately better/Much better
